# The Effect of the Traditional Mediterranean-Style Diet on Metabolic Risk Factors: A Meta-Analysis

**DOI:** 10.3390/nu8030168

**Published:** 2016-03-15

**Authors:** Marissa Garcia, Jessica D. Bihuniak, Julia Shook, Anne Kenny, Jane Kerstetter, Tania B. Huedo-Medina

**Affiliations:** 1 Department of Allied Health Sciences, University of Connecticut, Storrs, CT 06269, USA; Marissa.Garcia27@gmail.com (M.G.); Julia.shook@uconn.edu (J.S.); Jane.Kerstetter@uconn.edu (J.K.); 2 NYU Steinhardt, Department of Nutrition and Food Studies, New York University, New York, NY 10003, USA; jdb13@nyu.edu; 3 Center on Aging, University of Connecticut Health Center, Farmington, CT06030, USA; kenny@uchc.edu

**Keywords:** Mediterranean diet, metabolic syndrome, meta-analysis

## Abstract

The Mediterranean-style diet (MedSD) has gained attention for its positive effects on health outcomes, including metabolic risk factors. However, it is unknown as to which components of MedSD interventions are most beneficial in reducing risk. The objective of this meta-analysis was to obtain effect sizes for metabolic risk factors and explain the variability across the current literature based on study design, sample, and diet characteristics. Six electronic databases were searched from inception until 9 February 2016. Data from 29 studies (*N* = 4133) were included. There were significant effects in favor of the MedSD for waist circumference, triglycerides, blood glucose, systolic blood pressure, and diastolic blood pressure (*d*_+_ = −0.54; *d*_+_ = −0.46; *d*_+_ = −0.50; *d*_+_ = −0.72; *d*_+_ = −0.94, respectively). The MedSD was significantly beneficial when the intervention was longer in duration, was conducted in Europe, used a behavioral technique, and was conducted using small groups. The traditional MedSD had significant beneficial effects on five of the six metabolic risk factors. Results from this study provide support for population specific dietary guideline for metabolic risk reduction.

## 1. Introduction

Metabolic syndrome is defined as a group of interrelated risk factors of metabolic origin that appear to directly promote the development of cardiovascular disease (CVD) [1]. The National Cholesterol Education Program’s Adult Treatment Panel III report (NCEP ATPIII) [2] identified six components of metabolic syndrome that are related to CVD: (1) abdominal obesity; (2) atherogenic dyslipidemia; (3) elevated blood pressure; (4) insulin resistance; (5) proinflammatory state; and (6) prothrombotic state [2]. According to the ATP III criteria, a diagnosis of metabolic syndrome can be made when three out of five of the following characteristics are present: (1) abdominal obesity characterized by waist circumference (WC) >102 cm for men and >88 cm for women; (2) triglycerides (TG) ≥150 mg/dL; (3) HDL cholesterol (HDL) <40 mg/dL for men and <50 mg/dL for women; (4) blood pressure ≥130/≥85 mmHg; and (5) fasting glucose (FBG) ≥110 mg/dL [2]. Metabolic syndrome is a major health concern in the United States. Findings from the Third National Health and Nutrition Examination Survey (NHANES) suggest that according to the NCEP ATP III criteria approximately 34% of adults in the United States have metabolic syndrome [3].

Lifestyle therapies such as diet modification and physical activity are currently recommended as first-line interventions to reduce metabolic risk factors [1]. The Mediterranean-style diet (MedSD) is well-known for its cardio-protective benefits [4] and more recently, has been evaluated for the prevention and treatment of metabolic syndrome [5]. This dietary pattern emphasizes abundance of plant-based foods, a variety of minimally processed and locally grown foods, and olive oil as the principal source of fat [6]. The MedSD also includes daily consumption of low to moderate amounts of cheese and yogurt (low-fat and non-fat versions may be preferable), twice weekly consumption of fish and poultry, consumption of up to seven eggs per week, fresh fruit as dessert, red meat consumption limited to a few times a month, moderate consumption of wine (1 glass/day for women and 1–2 glasses/day for men) and regular physical activity at a level which promotes healthy weight and well-being [6].

To our knowledge, only one meta-analysis has evaluated literature on the effects of a MedSD on metabolic syndrome [7]. This meta-analysis included 35 clinical trials, two prospective studies, and 13 cross-sectional studies with a total of 534,906 participants and found an overall beneficial effect of the Mediterranean diet on reducing metabolic syndrome and its components in adults [7,8]. Further, the Scientific Report of the 2015 [8] found dietary characteristics similar to that of a MedSD, including higher intake of vegetables, fruits, seafood, legumes, and nuts; moderate intake of alcohol (among adults); lower consumption of red and processed meat, and low intake of sugar-sweetened foods and drinks [8], to have a positive effect on metabolic syndrome risk factors (*i.e.*, blood pressure and lipid profiles). Taken together, the findings from the meta-analysis by Kastorini *et al.* [7] noted above and the 2015 Advisory Committee on the Dietary Guidelines for Americans [8] clearly support the positive effects of the MedSD on metabolic risk factors. However, it is currently unclear which specific characteristics of MedSD-based interventions significantly contribute to the previously observed beneficial effects of a traditional MedSD on metabolic risk factors. We therefore conducted a high quality meta-analysis with specific attention to each criteria of metabolic syndrome, each component of the MedSD, and each methodological characteristic which may help to explain the difference in results between published studies.

## 2. Methods

### 2.1. Literature Search

The data sources were obtained following the Preferred Reporting Items for Systematic Reviews and Meta-Analyses: The PRISMA Statement [9] guidelines. Original research studies that were published regardless of publication type until 9 February 2016 were included. Language was not restricted. Six computer databases were searched: PubMed, EMBASE (via Scopus), Web of Science, CINAHL, Agricola, and CAB Direct. A comprehensive literature search was conducted with the assistance of the University of Connecticut Health Sciences Librarian (JL) using combinations of Medical Subject Headings and other key words related to the aim of the study. Examples of the key words include: “Mediterranean Diet”, “Mediterranean Style Diet”, adiposity, “metabolic syndrome”, overweight, BMI, “body mass”, “waist circumference”, obese, obesity, “abdominal fat”, and “weight loss”. The comprehensive search that was conducted for each database can be found in the supplemental material (S1). In addition to the electronic database search, all studies from Kastorini *et al.* [7] were screened and none of the studies overlap in both meta-analyses due to a difference in inclusion criteria.

### 2.2. Selection Criteria

**Figure 1:**
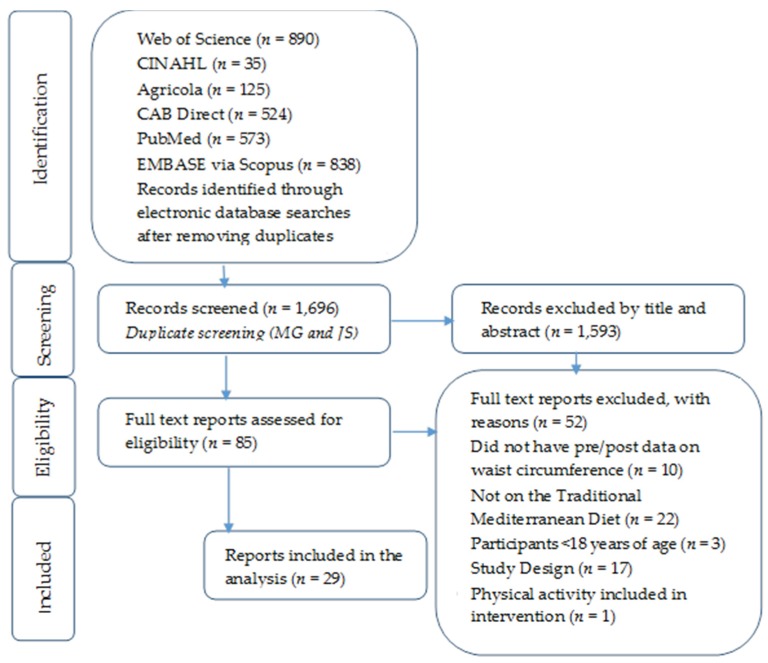
PRISMA Figure outlining the process of study identification, screening, eligibility, and inclusion.

Studies had to meet the following criteria to be included: (1) report pre-and post-intervention data on waist circumference (any other metabolic risk factors were additional); and (2) focus on the MedSD as a whole dietary pattern. Studies were excluded if they (1) did not have pre- and post-intervention data on waist circumference; (2) focused on particular components of the Mediterranean diet, such as only olive oil; (3) included exercise in the intervention; (4) included participants <18 years of age; (5) restricted calorie intake; and (6) did not report the information in a way that would allow effect sizes to be calculated using the published information. The relevance of studies was assessed by two independent researchers (M.G. and J.S.) with a hierarchical approach on the basis of title, abstract, and full manuscript. The original search resulted in 1696 abstracts with relevant key words. After screening and hand-searching articles, 29 articles (39 total comparisons) that used the traditional MedSD were included in analysis. Refer to Figure 1 for the PRISMA figure of included and excluded articles. A list of excluded articles is available in the supplemental material (Table S1).

### 2.3. Data Extraction

A comprehensive and detailed coding form and manual was created by a multidisciplinary team. The coding form includes approximately 330 variables for each study. Various characteristics were extracted from each study: (1) sample characteristics such as ethnicity, number and proportion of females, location of sample, and recruitment details; (2) intervention characteristics such as length of intervention, diet type, distribution of macronutrients, calorie intake, and participation in dietary counseling; and (3) study design characteristics such as number of interventions, type of control group, experimental conditions, and setting. The coding form was pilot-tested by two independent researchers (M.G. and J.S.) and was reviewed by additional experts (J.B., J.K., A.K., T.B.H.-M) before being finalized. The coding form and manual are available upon request to the corresponding author. All 30 studies were independently reviewed and coded by two researchers (M.G. and J.S.) and disagreements were solved by a third-party expert (T.B.H.-M).

### 2.4. Risk of Bias

The Cochrane Collaboration’s tool for assessing risk of bias was used to assess risk of bias within individual studies [10]. In accordance with these guidelines, we report descriptions of internal and external validity summary ratings categorically, converting these to numerical scores as necessary for the purpose of meta-analytic moderator analysis.

Methodological quality (MQ) rankings have been identified as an under-analyzed element of the data reported in meta-analyses [11]. In this meta-analysis, MQ ratings based on the Cochrane risk of bias scale were entered as one or more possible moderators into the mixed-effects meta-regression models.

### 2.5. Statistical Analysis

Inter-rater reliability was calculated for all continuous and categorical variables. The kappa (κ) coefficient was used to calculate categorical agreement [12] (kappa = 0.94, 96.9% agreement) and Pearson’s correlation coefficient was used to calculate continuous agreement [13] (*r* = 1). We tested for asymmetries by using the Begg [14], Egger [15], and trim-and-fill [16] statistical tests as well as the funnel plot [17] graphical technique. Publication bias, descriptive statistics, and reliability tests were calculated using R version 3.1.2 [18] and particularly, “metafor” package [19] for all the meta-analytic analysis.

Effect sizes (ESs) were calculated for each outcome by calculating the standardized mean change [20] for each sample [21], using the standard deviation of the pretest and adjusting by small sample sizes. The data extracted to obtain the individual ESs could be means and standard deviations, F-ANOVA, *t*-test, or mean and standard deviation change. To uphold the assumption of independence, each outcome was analyzed independently when multiple outcomes were reported from the same study. Twenty-two studies report at least three outcomes with the most common outcomes being waist circumference, HDL cholesterol, and triglycerides. Fourteen studies reported all six outcomes of interest. A multivariate approach for multiple subsamples per study was not followed because no more than five comparisons were available per study. Multiple ESs were obtained from the same study when data was reported separately by participant and diet characteristics [22,23]. Only two studies had subsamples based on sex [24,25] and three studies had multiple subsamples for participant characteristics [26,27,28].

Weighted mean effect size by the inverse of the variance of each study was calculated across all studies under random- and fixed-effects assumptions [29]. To test for heterogeneity, Cochran’s *Q* [30] and *I*^2^ [31] were calculated. To evaluate the sources of heterogeneity of the ESs, moderator analysis using weighted mixed-effects models with maximum likelihood estimation of the random-effects weights was performed testing each variable for study, intervention, and participant characteristics independently. The moving constant technique [32] was used to produce estimates of the ES (*d+*) at meaningful levels of the moderators and their Confidence Intervals (*Cis*) at different levels of interest. This technique was used to demonstrate results at the maximum and minimum values of significant moderators. Two-sided statistical significance was *p* < 0.05. Finally, clinical units of measures were included by transforming arithmetically the standardized ES to its unstandardized version [33].

## 3. Results

### 3.1. Description of Included Studies

A description of the included studies can be found in Table 1. Analysis of 29 reports shows that out of 4133 participants, 72% were female with a mean age of 46.93 (SD = 8.30). A majority of the studies were conducted in Europe (55.9%) and published in English (96.9%). Studies varied in design: 33.3% had a non-MedSD comparison group and 58.9% of studies were crossover or pre-/post-test only design. The mean publication year was 2009 (SD = 2.90) with a 12-year range from 2003 to 2015. The mean intervention length was 35.3 (SD = 50.71) weeks with a range from four to 208 weeks. No significant asymmetries were found using any of the statistical tests or the graphical funnel plot.

### 3.2. Effect Sizes

The traditional MedSD was found to have a significant beneficial effect on five out of six outcomes of interest (Table 2, Figures S1–S6). Overall ESs under random-effects assumptions indicate that the traditional MedSD had a significant overall effect on WC, TG, FBG, systolic blood pressure (SBP), and diastolic blood pressure (DBP) (*d_+_* = −0.54, 95% CI −0.77 to −0.31; *d_+_* = −0.46, 95% CI −0.72 to −0.21; *d_+_* = −0.50, 95% CI −0.81 to −0.20; *d_+_* = −0.72, 95% CI −1.03 to −0.42; *d_+_* = −0.94, 95% CI −1.45 to −0.44, respectively), but did not have a significant effect on HDL (*d_+_* = 0.19, 95% CI −0.07 to 0.46). There was large heterogeneity between studies with *I*^2^ ranging from 92.98% to 98.42%.

### 3.3. Moderator Analysis

All moderation effects are presented in Table 3. In regards to study characteristics, trending associations were found for study region. Studies conducted in Europe showed significant beneficial effects from the traditional MedSD intervention on four of the metabolic risk factors (waist circumference, HDL cholesterol, triglycerides and fasting blood glucose) whereas studies conducted in the United States did not result in significant effect sizes for any of the study characteristics.

Significant associations were found for study design waist circumference, HDL cholesterol, triglycerides, fasting blood glucose and systolic blood pressure. Studies that included a comparison intervention group design (*i.e.*, a different type of diet) had more beneficial significant effect sizes favoring the MedSD compared to those studies using a traditional pre-/post-design or a crossover design.

Studies with a higher *Impact per Publication* (IPP) value showed more significant beneficial effects for waist circumference, HDL cholesterol, triglycerides, and fasting blood glucose with significant positive associations for each.

The length of the intervention (in weeks) significantly explained between 27.89% and 51.13% of the variability between studies for the following outcomes: waist circumference, HDL cholesterol, triglycerides, fasting blood glucose and systolic blood pressure. There was a significant positive association for length of intervention for all six outcomes of interest (Figures S7–S11). The longer the length of the intervention, the more significant the beneficial effect in favor of the traditional MedSD. Additional significant or trending intervention characteristics included the use of a behavioral technique, supervision, and dietary interventions conducted primarily in small groups. The use of a behavioral technique resulted in trending or significant beneficial effects in all of the outcomes of interest compared to the effects when there was no behavioral technique used.

The level of intervention or supervision during the study (*i.e.*, primarily one-on-one or small groups) resulted in significant or trending associations for waist circumference, HDL cholesterol, triglycerides, fasting blood glucose and systolic blood pressure. Interventions consisting of small groups saw significant beneficial effects for all six outcomes, whereas interventions that were primarily one-on-one resulted in only two significant outcomes.

In regards to specific components of the traditional MedSD interventions, specific macronutrient proportions of the diet, assessment of dietary compliance and participant engagement in dietary counseling did not significantly explain the variability between studies. Participant characteristics, in particular the presence or absence of certain disease states, were also analyzed as moderators. Disease states that were included in this analysis were cardiovascular disease, type II diabetes mellitus, metabolic syndrome, and overweight/obesity. These variables were not considered to be significant moderators.

### 3.4. Risk of Bias

Risk of bias was unclear for random sequence generation, allocation, blinding, incomplete outcome data, selective reporting, and other potential sources of bias (Figure S12). Moderator analysis was not significant for any of the risk of bias parameters (data not shown). No high or low risk of bias was found for random sequence generation and 3.3% of the articles had low risk of bias for allocation concealment. As for blinding of participants and personnel, 6.7% of the articles had low risk of bias and 13.3% of the articles had high risk of bias. Blinding of outcome assessment had 10% low risk of bias and 10% high risk of bias. Incomplete outcome data in the short-term and long-term both resulted in 6.7% of articles with high risk of bias. No high or low risk of bias was reported for selective reporting. With regard to other bias, 3.3% of articles had low risk of bias whereas 10% had high risk of bias.

## 4. Discussion

The present meta-analysis of 29 intervention trials found that the traditional MedSD has significant beneficial effects for five out of six of the metabolic risk factors: waist circumference, triglycerides, fasting blood glucose, systolic blood pressure and diastolic blood pressure. The significant heterogeneity between studies was partly attributed to the location of the studies, the length of the intervention, and the IPP value of the journal where the study was published. Significant beneficial associations were found for studies conducted in Europe, those of longer duration, studies using a behavioral technique, studies with a comparison intervention group, and studies conducted primarily in groups for most of the metabolic risk factors. To our knowledge, this is the first meta-analysis to evaluate the effects of the Mediterranean diet on metabolic syndrome that meets 100% of the AMSTAR criteria [58].

Our findings that a traditional MedSD is beneficial in reducing the risk of CVD-associated metabolic parameters complements and extends previous work in this area. Several recent systematic reviews and meta-analyses published on the MedSD and CVD risk have reported similar positive effects on HDL cholesterol [59], triglycerides [59], systolic blood pressure [60,61], diastolic blood pressure [60,61], and fasting blood glucose [60]. These studies also found similar significant positive associations in moderator analysis for studies conducted in Mediterranean countries [7,62], duration of study [7], study design [62], and study quality [4,7].

To our knowledge, only one meta-analysis has been published on the effects of the Mediterranean diet on metabolic syndrome [7]. The meta-analysis by Kastorini *et al.* [7] included 35 clinical trials, two prospective studies, and 13 cross-sectional studies with a total of 534,906 participants. Consistent with our current analysis, Kastorini *et al.* found that the MedSD was associated with reductions in waist circumference, triglycerides and fasting glucose levels. The MedSD was also associated with beneficial effects on HDL cholesterol, whereas there were no association for systolic and diastolic blood pressure [7]. However, in the present meta-analysis we did not find a significant effect for HDL cholesterol and found a significant beneficial association for both systolic and diastolic blood pressure. The literature search employed by Kastorini *et al.* [7] differed from the current meta-analysis in that the search was limited to those manuscripts published in English and to three computer databases. Small literature searches of only a few key terms at a time were conducted rather than one comprehensive literature search. Clinical trials with lack of randomization, lack of a control diet group, and interventions without inclusion of all traditional Mediterranean diet components were excluded from their analysis [7]. For the present meta-analysis, a comprehensive literature search was performed using six electronic databases, language was not restricted and studies without comparison groups or with a lack of randomization were not excluded. Thus, differences in search criteria may have contributed to the reported discrepancies in the associations for the MedSD and HDL cholesterol and the MedSD and blood pressure between the present report and meta-analysis by Kastorini *et al.* [7].

### Study Limitation and Strengths

Our meta-analysis had several limitations. There is significant heterogeneity between studies that could not be explained by the moderators included in our analyses. The data reported in our sample of studies did not allow us to control for baseline physical activity or different types and duration of on-going exercise, and thus, physical activity could not be included as a moderator. Weight loss was not the objective in any of the included studies, however, we did not control for weight change among participants. Lastly, ecological fallacy is a possibility as we did not have access to the raw data from the included studies and should be cautious interpreting the group results as individual effects. There are also multiple strengths for this meta-analysis. We used a comprehensive literature search in six electronic databases and an inclusive and comprehensive coding form and manual were used for data extraction. We performed moderation analysis on all variables with sufficient data provided in the published material. We excluded interventions that included exercise, which we believed would have precluded us from solely evaluating diet-associated effects. To our knowledge, this is the first meta-analysis to find significant beneficial associations for MedSD interventions that use behavioral techniques and small group interventions and metabolic risk factors. Lastly, we were able to use the moving constant technique and a predictive model to calculate effect sizes for each significant moderator and transform that effect size into clinical units of measure.

## 5. Conclusions

The results of the present meta-analysis suggest that the traditional MedSD can have risk reduction effects on a number of metabolic parameters. In addition, the MedSD was significantly beneficial for different metabolic risk factors when, in general the intervention was longer in duration, the study was conducted in Europe, the report was published in a journal with higher Impact per Publication value, the study included a comparison intervention, a behavioral technique was used, and the study was conducted using small groups. More high-quality intervention studies conducted in non-European countries that control for physical activity and changes in weight, and include objective measures of compliance are warranted and would allow for further moderator analyses.

## Figures and Tables

**Table 1:** Description of Included Studies.

Study	Country	*N*	% F	Age	Diseases	Recruit-ment	Dietary Assessment	Type of Diet	Duration (Weeks)	Control	Outcome
Aizawa, *et al.* (2009) [26]	Canada	63	51%	53.9	PDM PHTN	Physician referral	Group, unsupervised	MedSD	24	No carotid artery stiffness	Carotid artery stiffness
Bedard, *et al.* (2012) [27]	Canada	67	NR	39	Ob (57%)	NR	Individual, supervised	MedSD	8	Non-Ob	CVDRF
Bekkouche, *et al.* (2014) [34]	Algeria	86	NR	52	MS (67%)	Hospital	Individual, unsupervised	MedSD	12	No MS, healthy	IR, OS, Inflam.
Bos, *et al.* (2010) [35]	Netherlands	60	NR	52.5	Ob (100%)	NR	Individual, unsupervised	MedSD	10	High SFA diet; High MUFA diet	Serum lipids, IS
Connolly, *et al.* (2011) [28]	Great Britain	206	42%	60.4	CVD or CVDRF (100%)	Hospital, physician referral	Individual, unsupervised	MedSD	16	None	CVDRF
Corbalan, *et al.* (2009) [36]	Spain	1406	82%	39	Ob (100%)	Clinic referral	Individual, unsupervised	MedSD	34	None	WT
Esposito, *et al.* (2006) [37]	Italy	65	0%	43.9	MS, ED (100%)	Research database	Individual, unsupervised	MedSD	24	Regular diet	IIEF score
Esposito, *et al.* (2007) [38]	Italy	59	100%	41.9	MS, FSD (100%)	Research Database	Individual, unsupervised	MedSD	24	Regular Diet	FSFI score
Esposito, *et al.* (2004) [39]	Italy	180	45%	43.9	MS (100%)	Clinic	Group, unsupervised	MedSD	104	Regular Diet	Endo func, Vas Infl
Esposito, *et al.* (2009) [40]	Italy	215	51%	52.2	NIDDM (100%)	Clinic	Group, unsupervised	MedSD	208	LF Diet	Glycemic control
Goulet, *et al.* (2003) [41]	Canada	77	100%	47	None, healthy	Newspaper ad.	Individual, unsupervised	MedSD	12	None	Serum lipid, WT
Goulet, *et al.* (2007) [42]	Canada	77	100%	46.7	None, healthy	Newspaper ad.	Individual, unsupervised	MedSD	24	None	WT
Jones, *et al.* (2011) [43]	United States	89	100%	47.5	MS (100%)	NR	Individual, unsupervised	MedSD-MF	12	MD, no MF	MS RF
Kolomvotsou, *et al.* (2013) [44]	Greece	90	48%	50.4	Ob (100%)	Hospital	Individual, unsupervised	MedSD	8	Regular diet	AO intake, plasma AO capacity
Leblanc, *et al.* (2014) [45]	Canada	108	47%	41.4	Ob, MS (100%)	Media advertise-ments	Individual and group, unsupervised and supervised	MedSD	12	None	Dietary intake, Met profile
Leighton, *et al.* (2009) [46]	Chile	145	0%	39	MS (24%)	Maestranza Diesel	Group, supervised	MedSD	52	None	MS RF
Lerman, *et al.* (2010) [47]	United States	24	83%	54.4	MS and high LDL-C (100%)	Previous study by Lerman	NR	MedSD-MF	12	MD, no MF	Plasma lipids
Lindeberg, *et al.* (2007) [48]	Sweden	29	0%	61	IHD, IGT, NIDDM	Hospital	Individual, unsupervised	MedSD	12	Paleolithic Diet	WT, serum glucose
Llaneza, *et al.* (2010) [49]	Spain	116	100%	56.4	IR (100%)	Hospital	Group, unsupervised	MedSD, soy supplement	104	MD, no supp	IR
Papandreou, *et al.* (2012 [50]	Greece	40	NR	41.5	Ob, OSAS (100%)	University Medical School	Group, unsupervised	MedSD	26	Prudent Diet	OSAS
Papandreou, *et al.* (2012) [51]	Greece	21	NR	41.5	Ob, OSAS (100%)	University Medical School	Group, unsupervised	MedSD	26	Prudent Diet	TBARS
Rallidis, *et al.* (2009) [52]	Greece	82	48%	50.4	Ob (100%)	Hospital	Individual, unsupervised	MedSD	8	Regular Diet	Endo func
Richard, *et al.* (2011) [53]	Canada	26	0%	49.4	MS (100%)	NR	Individual, unsupervised	MedSD	35	Western Diet	CVDRF
Rubenfire, *et al.* (2011) [24]	United States	126	68%	51	MS (100%)	Physician referral	Individual, unsupervised	MedSD	12	None	WT, BP, TG, serum glucose
Ryan, *et al.* (2013) [54]	Australia	12	50%	55	NAFLD (100%)	Hospital	Individual, unsupervised	MedSD	6	LF diet	WT, IS
Sanchez-Benito, *et al.* (2012) [55]	Spain	158	87%	48	OverWT (100%)	Pharmacy office	Individual, unsupervised	MedSD	26	None	BMI, BP, cholesterol
Stendall-Hollis, *et al.* (2013) [56]	United States	129	100%	29.7	OverWT (100%)	Magazine, hospital, Craigslist	Individual, unsupervised	MedSD	16	MyPyramid for P & B	WT, Inflam Bio
Timar, *et al.* (2013) [25]	Romania	223	50%	55	NIDDM (100%)	Diabetes Center	Group, unsupervised	MedSD	52	Diabetic Diet	Glycemic control, CVDRF
Van Velden, *et al.* (2007) [57]	South Africa	12	25%	46	MS (100%)	NR	Group, unsupervised	MedSD with red wine	8	MD without red wine	CVDRF

Note. *N*, number of participants at baseline; F, females; NR, not reported; OverWT, Overweight; Ob, Obesity; MedSD, Mediterranean Style Diet; PDM, Pre-diabetes mellitus; PHTN, Pre-hypertension; CVDRF, Cardiovascular Disease risk factors; MS, Metabolic Syndrome; OS, oxidative stress; NIDDM, Non-insulin Dependent Diabetes; IR, insulin resistance; Inflam, Inflammation; SFA, saturated fatty acid; IS, Insulin Sensitivity; ED, Erectile Dysfunction; IIEF, International Index of Erectile Function; FSD, Female Sexual Dysfunction; FSFI, Female Sexual Function Index; Endo Func., endothelial function; Vas Infl, vascular inflammation; MedSD-MF, Low-Glycemic Mediterranean Diet with Medical Food; MS RF, Metabolic Syndrome Risk Factors; AO, antioxidant; WC, waist circumference; IHD, ischaemic heart disease; IGT, impaired glucose tolerance; OSAS, Obstructive Sleep Apnea Syndrome; TBARS, thiobarbituric acid reacting substances; BP, blood pressure; TG, serum triglycerides; Inflam Bio, inflammatory biomarkers; MyPyramid for P&B, USDA MyPyramid Diet for Pregnant and Breastfeeding Women; FVII, activated factor VII; MI, Myocardial Infarction; Met profile, metabolic profile.

Note on Dietary Assessment column:
*Individual:* A dietitian performed a dietary assessment, providing individualized needs for caloric intake and recommendations, for each participant. *Group:* The study provided general dietary recommendations for the participants, such as a range of servings of certain food groups, calories based on sex, as opposed to tailoring diets to individual needs based on weight and height. *Supervised:* Participants consumed foods in a supervised setting, where the researchers had control over participant food choices and quantity of food served. *Unsupervised:* Participants food consumption was unsupervised by researchers, such as eating at home.

**Table 2:** Summary of Results, Overall Effect Sizes and Homogeneity.

Outcome	*k*	*d*_+_ (95% CI)		Homogeneity of *d*’s		
		Fixed-Effects	Random-Effects	*Q*	*I*^2^ (%)	*p*-Value
WC	39	−0.44 (−0.48 to −0.41) *	−0.54 (−0.77 to −0.31) *	390.1	96.39	<0.0001
HDL	27	0.15 (0.09 to 0.21) *	0.19 (−0.07 to 0.46)	294.6	93.95	<0.0001
TG	25	−0.34 (−0.40 to −0.28) *	−0.46 (−0.72 to −0.21) *	231.06	93.74	<0.0001
FBG	23	−0.37 (−0.42 to −0.33) *	−0.50 (−0.81 to −0.20) *	281.18	96.69	<0.0001
SBP	25	−0.74 (−0.78 to −0.70) *	−0.72 (−1.03 to −0.42) *	320.11	97.00	<0.0001
DBP	25	−0.99 (−1.06 to −0.93) *	−0.94 (−1.45 to −0.44) *	2263.05	98.42	<0.0001

Note: *d*_+_, overall effect size; WC, waist circumference; HDL, HDL cholesterol; TG, triglycerides, FBG, fasting blood glucose; SBP, systolic blood pressure; DBP, diastolic blood pressure; * indicates a significant effect; *k* represents the number of interventions for each outcome included in the analysis; *Q* represents Cochran’s *Q* indicating significance of heterogeneity; *I*^2^ represents the magnitude of heterogeneity; *p*-value represents the significance of heterogeneity.

**Table 3:** Significant Moderator Analysis Results.

Moderator	Outcome	Category	*k*	*d*_+_ (95% CI)	*R*^2^	*p*-Value	Clinical Unit of Measure
**Study Characteristics**							
Region	WC	Europe	19	−0.49 (−1.23 to 0.24)	2.25%	0.19	−1.23 cm
		US	7	−0.33 (−0.96 to 0.29)	2.90%	0.19	−0.83 cm
	HDL	Europe	10	0.80 (0.04 to 1.57)	19.3%	0.04	0.13 mmol/L
		US	6	−0.10 (−0.71 to 0.50)	19.3%	0.04	−0.02 mmol/L
	TG	Europe	9	−0.74 (−1.46 to −0.03)	4.11%	0.12	−0.35 mmol/L
		US	4	−0.13 (−073 to 0.46)	4.11%	0.12	−0.06 mmol/L
	FBG	Europe	9	−0.74 (−1.76 to 0.27)	9.14%	0.15	−0.06 mmol/L
		US	3	−0.18 (−1.05 to 0.69)	9.14%	0.15	−0.01 mmol/L
	SBP	Europe	10	−0.68 (−1.83 to 0.47)	0.36%	0.25	−3.21 mmol/L
		US	4	−0.47 (−1.44 to 0.50)	0.36%	0.25	−2.22 mmol/L
	DBP	Europe	10	−1.13 (−2.02 to 1.14)	2.88%	0.24	−3.47 mmol/L
		US	4	−0.44 (−1.95 to 0.74)	2.88%	0.24	−1.35 mmol/L
Study Design	WC	MedSD *vs.* Other Diet	13	−1.14 (−1.49 to −0.78)	28.71%	<0.0001	−2.87 cm
		Pre/Post or Crossover	23	−0.27 (−0.52 to −0.02)	28.71%	<0.0001	−0.68 cm
	HDL	MedSD *vs.* Other Diet	9	0.79 (0.45 to 1.15)	45.64%	<0.0001	0.13 mmol/L
		Pre/Post or Crossover	16	−0.16 (−0.42 to 0.09)	45.64%	<0.0001	−0.16 mmol/L
	TG	MedSD *vs.* Other Diet	8	−0.98 (−1.39 to −0.59)	28.04%	0.008	−0.46 mmol/L
		Pre/Post or Crossover	15	−0.21 (−0.49 to 0.07)	28.04%	0.008	−0.10 mmol/L
	FBG	MedSD *vs.* Other Diet	7	−1.13 (−1.59 to −0.66)	30.92%	<0.0001	−0.09 mmol/L
		Pre/Post or Crossover	14	−0.27 (−0.59 to 0.06)	30.92%	<0.0001	−0.02 mmol/L
	SBP	MedSD *vs.* Other Diet	7	−1.37 (−1.86 to −0.87)	32.26%	<0.0001	−6.47 mmHg
		Pre/Post or Crossover	16	−0.53 (−0.84 to −0.22)	32.26%	<0.0001	−2.69 mmHg
	DBP	MedSD *vs.* Other Diet	7	−1.32 (−2.31 to −0.32)	0.00%	0.004	−4.06 mmHg
		Pre/Post or Crossover	16	−0.87 (−1.52 to −0.21)	0.00%	0.004	−2.67 mmHg
Impact per Publication Metric	WC	0 (minimum)	39	−0.18 (−0.42 to 0.06)	50.13%	<0.0001	−0.45 cm
		16.104 (maximum)	39	−1.89 (−2.43 to −1.37)	50.13%	<0.0001	−4.76 cm
	HDL	0 (minimum)	26	−0.03 (−0.31 to 0.26)	29.44%	0.0006	−0.005 mmol/L
		16.104 (maximum)	26	0.95 (0.38 to 1.52)	29.44%	0.0006	0.15 mmol/L
	TG	0 (minimum)	24	−0.23 (−0.53 to 0.08)	22.65%	<0.0001	−0.11 mmol/L
		16.104 (maximum)	24	−1.09 (−1.68 to −0.52)	22.65%	<0.0001	−0.52 mmol/L
	FBG	0 (minimum)	22	−0.13 (−0.45 to 0.19)	41.52%	0.0004	−0.01 mmol/L
		16.104 (maximum)	22	−1.45 (−2.03 to −0.88)	41.52%	0.0004	−0.11 mmol/L
	SBP	0 (minimum)	24	−0.51 (−0.86 to −0.16)	13.10%	0.13	−2.41 mmHg
		16.104 (maximum)	24	−1.16 (−1.84 to −0.49)	13.10%	0.13	−5.48 mmHg
	DBP	0 (minimum)	23	−0.69 (−1.37 to 0.02)	3.54%	0.18	−2.12 mmHg
		16.104 (maximum)	23	−1.77 (−3.06 to −0.49)	3.54%	0.18	−5.44 mmHg
**Intervention Characteristics**							
Length of intervention (in weeks)	WC	4 weeks (minimum)	39	−0.24 (−0.45 to −0.03)	46.18%	<0.0001	−0.604 cm
		208 weeks (maximum)	39	−2.50 (−3.29 to −1.71)	46.18%	<0.0001	−6.29 cm
	HDL	4 weeks (minimum)	27	−0.09 (−0.32 to 0.14)	48.04%	<0.0001	−0.01 mmol/L
		208 weeks (maximum)	27	1.79 (1.06 to 2.53)	48.04%	<0.0001	0.29 mmol/L
	TG	4 weeks (minimum)	25	−0.19 (−0.46 to 0.07)	32.1%	0.0009	−0.09 mmol/L
		208 weeks (maximum)	25	−1.73 (−2.51 to −0.95)	32.1%	0.0009	−0.83 mmol/L
	FBG	4 weeks (minimum)	23	−0.19 (−0.45 to 0.07)	51.13%	<0.0001	−0.01 mmol/L
		208 weeks (maximum)	23	−2.22 (−3.02 to −1.41)	51.1%	<0.0001	−0.17 mmol/L
	SBP	4 weeks (minimum)	25	−0.45 (−0.77 to −0.14)	27.89%	0.0004	−2.12 mmHg
		208 weeks (maximum)	25	−2.04 (−2.98 to −1.09)	27.89%	0.004	−9.63 mmHg
	DBP	4 weeks (minimum)	25	−0.67 (−1.26 to −0.08)	6.39%	0.10	−2.06 mmHg
		208 weeks (maximum)	25	−2.37 (−4.15 to −0.59)	6.39%	0.10	−7.28 mmHg
Number of Females	WC	0 (minimum)	35	−0.49 (−0.76 to −0.23)	0.00%	0.95	−1.23 cm
		1154 (maximum)	35	−0.54 (−1.91 to 0.83)	0.00%	0.95	−1.36 cm
	HDL	0 (minimum)	25	0.33 (−0.06 to 0.72)	0.00%	0.39	0.06 mmol/L
		1154 (maximum)	25	−3.43 (−11.69 to 4.83)	0.00%	0.39	−0.56 mmol/L
	TG	0 (minimum)	23	−0.45 (−0.87 to −0.03)	0.00%	0.89	−0.22 mmol/L
		1154 (maximum)	23	−1.04 (−10.91 to 8.83)	0.00%	0.91	−0.49 mmol/L
	FBG	0 (minimum)	21	−0.55 (−0.91 to −0.19)	0.00%	0.91	−0.04 mmol/L
		1154 (maximum)	21	−0.46 (−1.94 to 1.01)	0.00%	0.91	−0.04 mmol/L
	SBP	0 (minimum)	23	−0.70 (−1.04 to −0.36)	0.00%	0.79	−3.31 mmHg
		1154 (maximum)	23	−0.91 (−2.36 to 0.53)	0.00%	0.79	−4.29 mmHg
	DBP	0 (minimum)	22	−0.59 (−0.95 to −0.25)	67.92%	<0.0001	−1.81 mmHg
		1154 (maximum)	22	−5.82 (−7.29 to −4.33)	67.92%	<0.0001	−17.89 mmHg
Total sample size	WC	12 (minimum)	39	−0.54 (−0.81 to −0.26)	0.00%	0.97	−1.36 cm
		1406 (maximum)	39	−0.56 (−1.88 to 0.77)	0.00%	0.97	−1.41 cm
	HDL	12 (minimum)	27	−0.18 (−0.63 to 0.27)	13.29%	0.05	−0.03 mmol/L
		1406 (maximum)	27	5.69 (0.10 to 11.29)	13.29%	0.05	0.93 mmol/L
	TG	12 (minimum)	25	−0.20 (−0.66 to 0.26)	4.55%	0.18	−0.09 mmol/L
		1406 (maximum)	25	−4.65 (−10.79 to 1.48)	4.55%	0.18	−2.22 mmol/L
	FBG	12 (minimum)	23	−0.49 (−0.84 to −0.15)	0.00%	0.85	−0.04 mmol/L
		1406 (maximum)	23	−0.64 (−2.05 to −0.78)	0.00%	0.85	−0.05 mmol/L
	SBP	12 (minimum)	25	−0.71 (−1.05 to −0.35)	0.00%	0.79	−3.35 mmHg
		1406 (maximum)	25	−0.93 (−2.38 to 0.53)	0.00%	0.79	−4.39 mmHg
	DBP	12 (minimum)	24	−0.41 (−0.73 to −0.09)	72.14%	<0.0001	−1.26 mmHg
		1406 (maximum)	24	−5.9 (−7.22 to −4.58)	72.14%	<0.0001	−18.14 mmHg
Sample size of intervention group	WC	11 (minimum)	39	−0.54 (−0.79 to −0.28)	0.00%	0.99	−1.36 cm
		1154 (maximum)	39	−0.55 (−1.93 to 0.82)	0.00%	0.99	−1.38 cm
	HDL	11 (minimum)	27	0.11 (−0.33 to 0.54)	0.00%	0.60	0.02 mmol/L
		1154 (maximum)	27	2.24 (−5.41 to 9.89)	0.00%	0.60	0.37 mmol/L
	TG	11 (minimum)	25	−0.34 (−0.76 to 0.07)	0.00%	0.47	−0.16 mmol/L
		1154 (maximum)	25	−3.45 (−11.62 to 4.73)	0.00%	0.47	−1.65 mmol/L
	FBG	11 (minimum)	23	−0.50 (−0.84 to −0.17)	0.00%	0.96	−0.04 mmol/L
		1154 (maximum)	23	−0.54 (−1.96 to 0.88)	0.00%	0.96	−0.04 mmol/L
	SBP	11 (minimum)	25	−0.71 (−1.05 to −0.37)	0.00%	0.78	−3.35 mmHg
		1154 (maximum)	25	−0.93 (−2.39 to 0.53)	0.00%	0.78	−4.39 mmHg
	DBP	11 (minimum)	24	−0.51 (−0.82 to −0.20)	71.80%	<0.0001	−1.57 mmHg
		1154 (maximum)	24	−5.91 (−7.25 to −4.58)	71.80%	<0.0001	−18.17 mmHg
Use of a behavioral technique	WC	No	21	−0.43 (−0.74 to −0.11)	0.00%	<0.0001	−1.08 cm
		Yes	18	−0.66 (−1.00 to −0.33)	0.00%	<0.0001	−1.66 cm
	HDL	No	14	−0.08 (−0.42 to 0.26)	13.88%	0.02	−0.01 mmol/L
		Yes	13	0.48 (0.13 to 0.83)	13.88%	0.02	0.08 mmol/L
	TG	No	14	−0.27 (−0.61 to 0.06)	6.26%	0.0003	−0.13 mmol/L
		Yes	11	−0.70 (−1.08 to −0.33)	6.26%	0.0003	−0.33 mmol/L
	FBG	No	12	−0.29 (−0.71 to 0.12)	4.51%	0.001	−0.02 mmol/L
		Yes	11	−0.72 (−1.14 to −0.29)	4.51%	0.001	−0.06 mmol/L
	SBP	No	13	−0.53 (−0.94 to −0.12)	1.71%	<0.0001	−2.50 mmHg
		Yes	12	−0.94 (−1.37 to −0.51)	1.71%	<0.0001	−4.44 mmHg
Level of intervention or supervision during the study	WC	Primarily one-on-one	14	−0.47 (−0.83 to −0.11)	17.28%	<0.0001	−1.18 cm
		Small groups	9	−1.14 (−1.58 to −0.69)	17.28%	<0.0001	−2.87 cm
	HDL	Primarily one-on-one	8	−0.18 (−0.63 to 0.28)	15.73%	0.03	−0.03 mmol/L
		Small groups	9	0.65 (0.23 to 1.07)	15.73%	0.03	0.11 mmol/L
	TG	Primarily one-on-one	8	−0.14 (−0.55 to 0.27)	16.04%	<0.0001	−0.07 mmol/L
		Small groups	7	−1.03 (−1.45 to −0.59)	16.04%	<0.0001	−0.49 mmol/L
	FBG	Primarily one-on-one	7	−0.19 (−0.69 to 0.32)	19.26%	0.0002	−0.01 mmol/L
		Small groups	7	−1.04 (−1.54 to −0.55)	19.26%	0.0002	−0.08 mmol/L
	SBP	Primarily one-on-one	9	−0.48 (−0.92 to −0.04)	28.65%	<0.0001	−2.26 mmHg
		Small groups	7	−1.43 (−1.93 to −0.94)	28.65%	<0.0001	−6.75 mmHg
	DBP	Primarily one-on-one	9	−0.37 (−1.19 to 0.46)	2.44%	0.002	−1.13 mmHg
		Small groups	7	−1.54 (−2.48 to −0.60)	2.44%	0.002	−4.73 mmHg

Note: WC, waist circumference; HDL, HDL cholesterol; TG, triglycerides, FBG, fasting blood glucose; SBP, systolic blood pressure; DBP, diastolic blood pressure; k is the number of interventions included in the analysis for each outcome; *R*^2^ indicates the percentage of heterogeneity that the moderator accounts for; *p*-value represents the significance of the moderation effect; Clinical Unit of Measure was calculated using a predictive model transforming arithmetically the standardized ES to its unstandardized version.

## Supplementary Materials

Supplementary Materials are available online at http://www.mdpi.com/2072-6643/8/3/168/s1.

## Abbreviations

The following abbreviations are used in this manuscript:

MedSD	Mediterranean-style Diet
CVD	Cardiovascular Disease
PRISMA	Preferred Reporting Items for Systematic Reviews and Meta-Analyses
MQ	Methodological Quality
ESs	Effect Sizes
WC	Waist circumference
FBG	Fasting blood glucose
TG	Triglycerides
SBP	Systolic blood pressure
DBP	Diastolic blood pressure
IPP	Impact per publication
