# Supplementary material for: The Effect of the Traditional Mediterranean-Style Diet on Metabolic Risk Factors: A Meta-Analysis
Source: Nutrients. 2016 Mar 15;8(3):168. doi: 10.3390/nu8030168 (PMC4808896; doi:10.3390/nu8030168)
Supplement: Supplementary file 1 [file nutrients-08-00168-s001.docx]

Supplemental Material: The Effect of the Traditional Mediterranean-Style Diet on Metabolic Risk Factors: A Meta-Analysis

Marissa Garcia, Jessica D. Bihuniak, Julia Shook, Anne Kenny, Jane Kerstetter
and Tania B. Huedo-Medina

S1. Comprehensive Literature Search Strategy

Databases searched until 9 February 2016 unless otherwise noted.

1. PubMed, years 1940–present

Terms were searched in all fields; however, field labels were used to restrict specific terms/phrases to the Medical Subject Headings [Mesh], publication type [pt] and journal name [ta] fields.

(“Mediterranean diet” OR “Mediterranean diets” OR “Mediterranean dietary” OR
“Mediterranean style diet” OR “Mediterranean style diets” OR “Diet,
Mediterranean”[Mesh]) AND (adiposity OR “metabolic syndrome” OR overweight OR
BMI OR “body mass” OR “waist circumference” OR weight [tiab] OR “body weight” OR
obese OR obesity OR “abdominal fat” OR “Weight Loss”[Mesh] OR “weight loss” OR
“Diet, Reducing”[Mesh]) NOT (“Cross-Sectional Studies”[MeSH Terms] OR “Case Reports”[pt] OR Comment[pt] OR Editorial[pt] OR Letter[pt] OR Review[pt] OR “case control”[ti] OR “case report”[ti] OR “case study”[ti] OR “case series”[ti] OR “Case-Control Studies”[Mesh] OR
“Follow-Up Studies”[Mesh] OR “observational study”[ti] OR “prospective cohort”[ti] OR
“cohort studies”[Mesh:noexp] OR “cohort study”[ti] OR “Longitudinal Studies”[Mesh:noexp] OR “Follow-Up Studies”[mesh] OR “Retrospective Studies”[mesh] OR “non-randomized”[ti] OR
“follow up study”[ti] OR rat[ti] OR rats[ti] OR mice[ti] OR mouse[ti] OR dog[ti] OR dogs[ti] OR cats[ti])

Results: 573

2. EMBASE (via Scopus) years 1823–present

All terms were searched in “Article Title, Abstract, Keywords”. Because of character restrictions in Scopus, this search was run in parts and assembled using the “Search history”.

Limit to Document type: “Article”

{Mediterranean diet} OR {Mediterranean diets} OR {Mediterranean dietary} OR {Mediterranean style diet} OR {Mediterranean style diets}

AND (adiposity OR {weight loss} OR {metabolic syndrome} OR overweight OR BMI OR
{body mass} OR {waist circumference} OR weight OR {body weight} OR obese OR
obesity OR {abdominal fat})

NOT (in article title) ({Cross-Sectional Studies} OR {Case Reports} OR Comment OR
Editorial OR Letter OR Review OR {case control} OR {case report} OR {case study} OR
{case series} OR {Follow-Up Study} OR {observational study} OR {prospective cohort} OR
{cohort study} OR {Longitudinal Study} OR {Follow-Up Studies} OR {Retrospective Studies} OR
{non-randomized} OR {follow up study} OR rat OR rats OR mice OR mouse OR dog OR
dogs OR cats)

Results: 838

3. Web of Science, years 1974-August 4, 2014 (The University of Connecticut discontinued its subscription to Scopus prior to the time of revisions in favor of other alternative resources, including Scopus)

All terms were searched in “Topic”.

Limit to Document type: “article”

“Mediterranean diet” OR “Mediterranean diets” OR “Mediterranean dietary” OR “Mediterranean style diet” OR “Mediterranean style diets”

AND

(adiposity OR “weight loss” OR “metabolic syndrome” OR overweight OR BMI OR “body mass” OR “waist circumference” OR weight OR “body weight” OR obese OR obesity OR “abdominal fat”)

NOT (in title) (“Cross-Sectional Studies” OR “Case Reports” OR Comment OR Editorial OR Letter OR Review OR “case control” OR “case report” OR “case study” OR “case series” OR “Follow-Up Study” OR “observational study” OR “prospective cohort” OR “cohort study” OR “Longitudinal Study” OR “Follow-Up Studies” OR “Retrospective Studies” OR “non-randomized” OR “follow up study” OR rat OR rats OR mice OR mouse OR dog OR dogs OR cats)

Results: 890

4. CINAHL

All terms were searched in all fields.

Excluded: MEDLINE Records

Limited to: research articles

“Mediterranean diet” OR “Mediterranean diets” OR “Mediterranean dietary” OR “Mediterranean style diet” OR “Mediterranean style diets”

AND

(adiposity OR “weight loss” OR “metabolic syndrome” OR overweight OR BMI OR “body mass” OR “waist circumference” OR weight OR “body weight” OR obese OR obesity OR “abdominal fat”)

NOT (in title) (“Cross-Sectional Studies” OR “Case Reports” OR Comment OR Editorial OR Letter OR Review OR “case control” OR “case report” OR “case study” OR “case series” OR “Follow-Up Study” OR “observational study” OR “prospective cohort” OR “cohort study” OR “Longitudinal Study” OR “Follow-Up Studies” OR “Retrospective Studies” OR “non-randomized” OR “follow up study” OR rat OR rats OR mice OR mouse OR dog OR dogs OR cats)

Results: 35

5. Agricola years 1970-present

Searched in “All Fields”

Limited to “academic journals”

“Mediterranean diet” OR “Mediterranean diets” OR “Mediterranean dietary” OR “Mediterranean style diet” OR “Mediterranean style diets”

AND

(adiposity OR “weight loss” OR “metabolic syndrome” OR overweight OR BMI OR “body mass” OR “waist circumference” OR weight OR “body weight” OR obese OR obesity OR “abdominal fat”)

NOT (in title) (“Cross-Sectional Studies” OR “Case Reports” OR Comment OR Editorial OR Letter OR Review OR “case control” OR “case report” OR “case study” OR “case series” OR “Follow-Up Study” OR “observational study” OR “prospective cohort” OR “cohort study” OR “Longitudinal Study” OR “Follow-Up Studies” OR “Retrospective Studies” OR “non-randomized” OR “follow up study” OR rat OR rats OR mice OR mouse OR dog OR dogs OR cats)

Results: 125

6. CAB Direct years 1973-present

Limit to Document Type: Journal article

“Mediterranean diet” OR “Mediterranean diets” OR “Mediterranean dietary” OR “Mediterranean style diet” OR “Mediterranean style diets”

AND

(adiposity OR “weight loss” OR “metabolic syndrome” OR overweight OR BMI OR “body mass” OR “waist circumference” OR weight OR “body weight” OR obese OR obesity OR “abdominal fat”)

NOT (in title) (“Cross-Sectional Studies” OR “Case Reports” OR Comment OR Editorial OR Letter OR Review OR “case control” OR “case report” OR “case study” OR “case series” OR “Follow-Up Study” OR “observational study” OR “prospective cohort” OR “cohort study” OR “Longitudinal Study” OR “Follow-Up Studies” OR “Retrospective Studies” OR “non-randomized” OR “follow up study” OR rat OR rats OR mice OR mouse OR dog OR dogs OR cats)

Results: 524

TOTAL: 1,696 after removal of duplicates

**Table S1.** Table of Excluded Articles.

| **Author** | **Title** | **Reason** |
| --- | --- | --- |
| Abenavoli, *et al.* | Effects of Mediterranean diet supplemented with silybin-vitamin E-phospholipid complex in overweight patients with non-alcoholic fatty  liver disease | Prospective study |
| Alacid, *et al.* | Habit based consumptions in the Mediterranean diet and the relationship with anthropometric parameters in young female kayakers | Not an intervention |
| Ambring, *et al.* | Effects of a Mediterranean-inspired diet on blood lipids, vascular function and oxidative stress in healthy subjects | Not on the traditional Mediterranean diet |
| Andreoli, *et al.* | Effect of a moderately hypoenergetic Mediterranean diet and exercise program on body cell mass and cardiovascular risk factors in obese women | Not on the traditional Mediterranean diet |
| Aronis, *et al.* | Effect of fast-food Mediterranean-type diet on human plasma oxidation | Not on the traditional Mediterranean diet |
| Athyros, *et al.* | Effect of a plant stanol ester-containing spread, placebo spread, or Mediterranean diet on estimated cardiovascular risk and lipid, inflammatory and haemostatic factors | Does not measure WC |
| Babio, *et al.* | Adherence to the Mediterranean diet and risk of metabolic syndrome and its components. | Cross sectional study |
| Barchitta, *et al.* | Tumor necrosis factor-alpha-308 g >a polymorphism, adherence to Mediterranean diet, and risk of overweight/obesity in young women | Did not report data on an outcome of interest |
| Barzi, *et al.* | Mediterranean diet and all-causes mortality after myocardial infarction: results from the GISSIPrevenzione trial | Observational  cohort study |
| Bedard, *et al.* | The Impact of Abdominal Obesity Status on Cardiovascular Response to the Mediterranean Diet | Not on the traditional Mediterranean diet |
| Bedard, *et al.* | Effects of a dietary intervention promoting the adoption of a Mediterranean food pattern on fast-food consumption among healthy  French-Canadian women | Not on the traditional Mediterranean diet |
| Bellido, *et al.* | The Mediterranean and CHO diets decrease VCAM-1 and E-selectin expression induced by modified low-density lipoprotein in HUVECs | Does not measure WC |
| Bonfanti, *et al.* | Effect of two hypocaloric diets and their combination with physical exercise on basal metabolic rate and body composition | Not on the traditional Mediterranean diet |
| Bravo-Herrara, *et al.* | Tissue factor expression is decreased in monocytes obtained from blood during Mediterranean or high carbohydrate diets | Does not measure WC |
| Buscemi, *et al.* | Effects of hypocaloric very-low-carbohydrate diet *vs.* Mediterranean diet on endothelial function in obese women | Does not report post intervention data for measures of obesity |
| Calatylud, *et al.* | Effects of the Mediterranean traditional diet in overweight and obese children after one year of intervention | Subjects <18 years  of age |
| Castaner, *et al.* | In vivo transcriptomic profile after a Mediterranean diet in high-cardiovascular risk patients:  a randomized controlled trial | Not on the traditional Mediterranean diet; compares olive oil and nut consumption |
| Castro, *et al.* | Comparison of an oleic acid enriched-diet *vs.*  NCEP-I diet on LDL susceptibility to  oxidative modi^®^cations | Not on the traditional Mediterranean diet |
| Cordero, *et al.* | The Guadix study of the effects of a Mediterranean-diet breakfast on the postprandial lipid parameters of overweight and obese pre-adolescents | Subjects <18 years  of age |
| Damasceno, *et al.* | Mediterranean diet supplemented with nuts reduces waist circumference and shifts lipoprotein subfractions to a less atherogenic pattern in subjects at high cardiovascular risk | Not on the traditional Mediterranean diet; compares olive oil and nut consumption |
| de Loregril, *et al.* | Mediterranean alpha-linolenic acid-rich diet in secondary prevention of coronary heart disease. | Prospective study |
| Del Mar Bibiloni, *et al.* | Compliance with the Mediterranean Diet Quality Index (KIDMED) among Balearic Islands' Adolescents and Its Association with Socioeconomic, Anthropometric and  Lifestyle Factors | Cross sectional study |
| Elhayany, *et al.* | A low carbohydrate Mediterranean diet improves cardiovascular risk factors and diabetes control among overweight patients with type 2 diabetes mellitus: a 1-year prospective randomized intervention study. | Prospective study |
| Estruch, *et al.* | Effects of dietary fibre intake on risk factors for cardiovascular disease in subjects at high risk | Not on the traditional Mediterranean diet; compares olive oil and nut consumption |
| Estruch, *et al.* | Effects of a Mediterranean-style diet on cardiovascular risk factors: a randomized trial | Not on the traditional Mediterranean diet; compares olive oil and nut consumption |
| Fernandez, *et al.* | Moderate-to-high-intensity training and a hypocaloric Mediterranean diet enhance endothelial progenitor cells and fitness in subjects with the metabolic syndrome | Not on the traditional Mediterranean diet |
| Fito, *et al.* | Effect of the Mediterranean diet on heart failure biomarkers: a randomized sample from the PREDIMED trial | Not on the traditional Mediterranean diet; compares olive oil and nut consumption |
| Fuentes, *et al.* | Mediterranean and Low-Fat Diets Improve Endothelial Function in Hypercholesterolemic Men | Does not measure WC |
| Garcia-Hermoso, *et al.* | Abdominal obesity as a mediator of the influence of physical activity on insulin resistance in  Spanish adults | Cross sectional study |
| Gardener, *et al.* | Mediterranean-style diet and risk of ischemic stroke, myocardial infarction, and vascular death: The Northern Manhattan Study | Cohort study |
| Gerhard, *et al.* | Effects of a low-fat diet compared with those of a high-monounsaturated fat diet on body weight, plasma lipids and lipoproteins, and glycemic control in type 2 diabetes. | Intervention not defined as a traditional Mediterranean diet |
| Gillingham, *et al.* | Dietary monounsaturated fatty acids are protective against metabolic syndrome and cardiovascular disease risk factors | Review |
| Gomez, *et al.* | Effect of the Mediterranean Diet on Fasting Concentrations of Activated Factor VII in  Healthy Persons | Does not measure WC |
| Gouveri, *et al.* | Mediterranean diet and metabolic syndrome in an urban population: The Athens study | Cross sectional study |
| Hermsdorff, *et al.* | Discriminated benefits of a Mediterranean dietary pattern within a hypocaloric diet program on plasma RBP4 concentrations and other inflammatory markers in obese subjects | Not on the traditional Mediterranean diet |
| Jula, *et al.* | Effects of Diet and Simvastatin on Serum  Lipids, Insulin, and Antioxidants in Hypercholesterolemic Men | Does not measure WC |
| Karatonis, *et al.* | Effect of fast-food Mediterranean-type diet on type 2 diabetics and healthy human subjects’  platelet aggregation | Not on the traditional Mediterranean diet |
| Katz, *et al.* | Effects of Walnuts on Endothelial Function in Overweight Adults with Visceral Obesity:  A Randomized, Controlled, Crossover Trial | Not on the traditional Mediterranean diet; focusing only on the effect of one component walnuts |
| Landaeta-Diaz, *et al.* | Mediterranean diet, moderate-to-high intensity training, and health-related quality of life in adults with metabolic syndrome | Physical activity included |
| McManus, *et al.* | A randomized controlled trial of a moderate-fat, low-energy diet compared with a low fat,  low-energy diet for weight loss in  overweight adults. | Prospective study |
| Mendez, *et al.* | Effectiveness of a nutrition intervention in patients with hypertension in primary health care: results of a pilot study in Mexico City | Does not measure WC; includes physical activity |
| Paniagua, *et al.* | A MUFA-Rich Diet Improves Posprandial Glucose, Lipid and GLP-1 Responses in Insulin-Resistant Subjects | Prospective study |
| Paraskevas, *et al.* | Definition of Best Medical Treatment in Asymptomatic and Symptomatic Carotid  Artery Stenosis | Review |
| Perez-Jimenez, *et al.* | A Mediterranean and a high-carbohydrate diet improve glucose metabolism in healthy  young persons | Does not measure WC |
| Perez-Martinez, *et al.* | The chronic intake of a Mediterranean diet enriched in virgin olive oil, decreases nuclear transcription factor kappaB activation in peripheral blood mononuclear cells from healthy men | Does not measure WC |
| Piers, *et al.* | Substitution of saturated with monounsaturated fat in a 4-week diet affects body weight and composition of overweight and obese men. | Not on the traditional Mediterranean diet |
| Rodriguez Villar, *et al.* | Comparison of a high-carbohydrate and a  high-monounsaturated fat, olive oil-rich diet on the susceptibility of LDL to oxidative modification-tion in subjects with Type 2 diabetes mellitus | Not on the traditional Mediterranean diet |
| Salas-Salvado, *et al.* | Effect of a Mediterranean diet supplemented with nuts on metabolic syndrome status: one-year results of the PREDIMED randomized trial. | Not on the traditional Mediterranean diet; compares olive oil and nut consumption |
| Singh, *et al.* | Effects of a “healthy” diet and of acute and long-term vitamin C on vascular function in healthy older subjects. | Not on the traditional Mediterranean diet |
| Sondergaaud, *et al.* | Effect of dietary intervention and lipid-lowering treatment on brachial vasoreactivity in patients with ischemic heart disease and hypercholesterolemia. | Does not measure WC |
| Velazquez-Lopez, *et al.* | Mediterranean-style diet reduces metabolic syndrome components in obese children and adolescents with obesity | Subjects <18 years  of age |
| Wardle, *et al.* | Randomized Trial of the Effects of  Cholesterol-lowering Dietary Treatment on Psychological Function | Does not measure WC |
| Zazpe, *et al.* | Association between dietary carbohydrate intake quality and micronutrient intake adequacy in a Mediterranean cohort: The SUN (Seguimiento Universidad de Navarra) project | Cohort study |


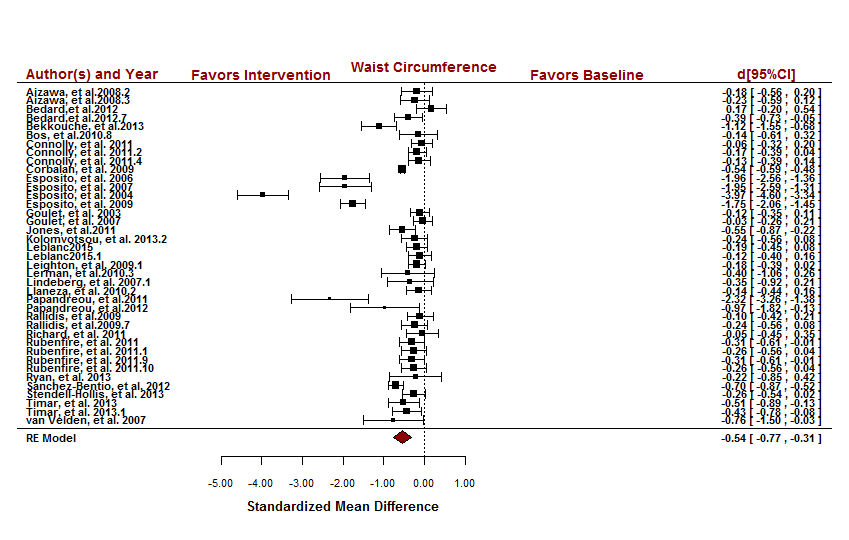


**Figure S1.** Forest Plot for Waist Circumference. Note: Squares represent point estimates for each individual study; extended line shows 95% confidence intervals (CIs); dotted line represents the null value of zero; diamond represents the weighted mean effect size for the outcome.


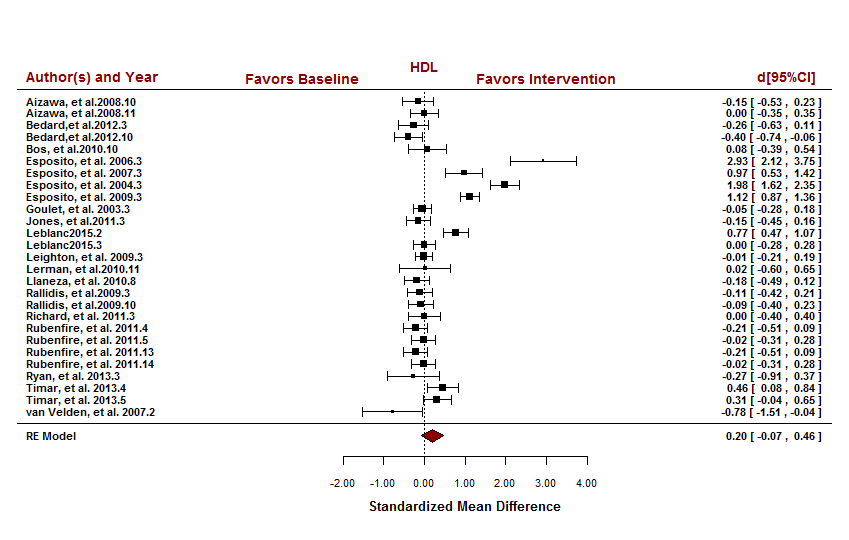


**Figure S2.** Forest Plot for HDL. Note: Squares represent point estimates for each individual study; extended line shows 95% confidence intervals (CIs); dotted line represents the null value of zero; diamond represents the weighted mean effect size for the outcome.


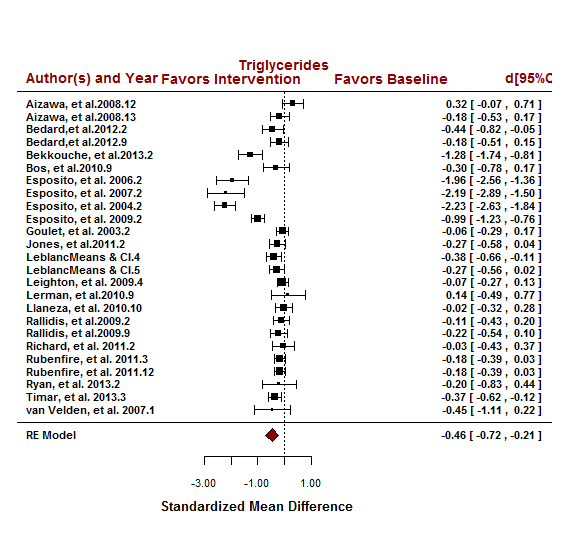


**Figure S3.** Forest Plot for Triglycerides. Note: Squares represent point estimates for each individual study; extended line shows 95% confidence intervals (CIs); dotted line represents the null value of zero; diamond represents the weighted mean effect size for the outcome.


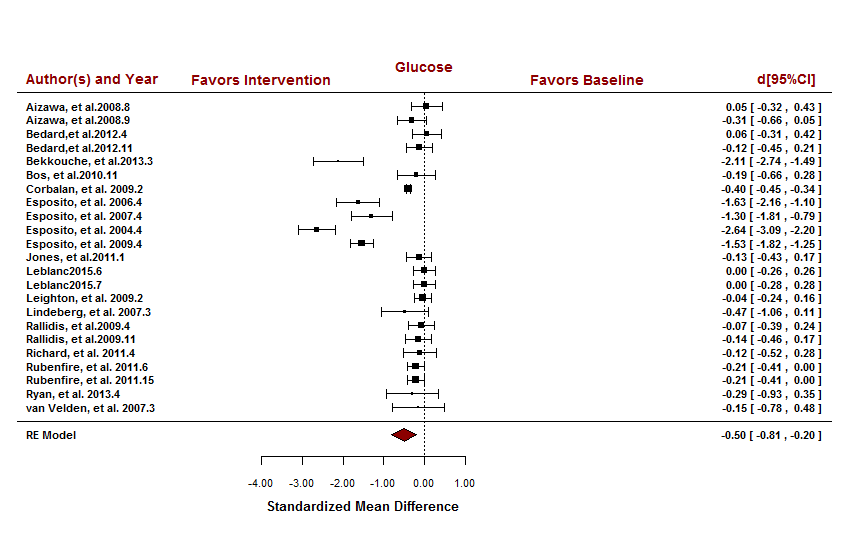


**Figure S4.** Forest Plot for Fasting Blood Glucose. Note: Squares represent point estimates for each individual study; extended line shows 95% confidence intervals (CIs); dotted line represents the null value of zero; diamond represents the weighted mean effect size for the outcome.


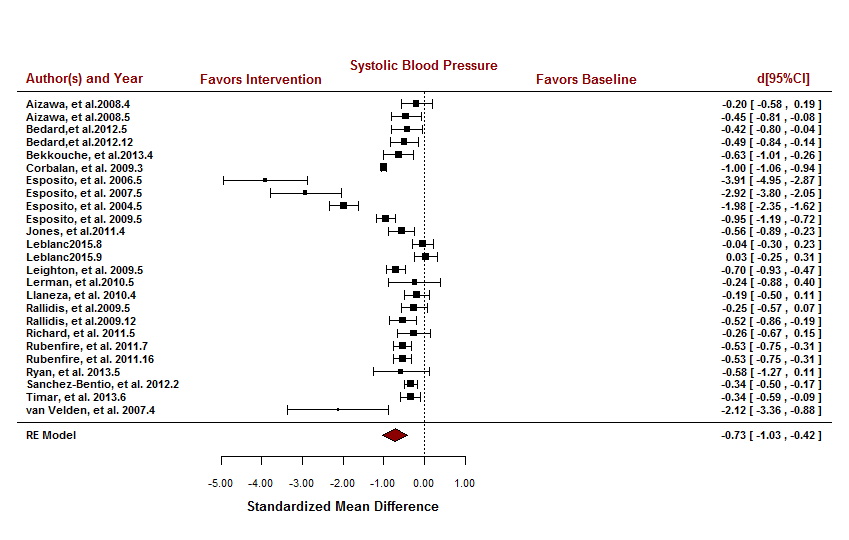


**Figure S5.** Forest Plot for Systolic Blood Pressure. Note: Squares represent point estimates for each individual study; extended line shows 95% confidence intervals (CIs); dotted line represents the null value of zero; diamond represents the weighted mean effect size for the outcome.


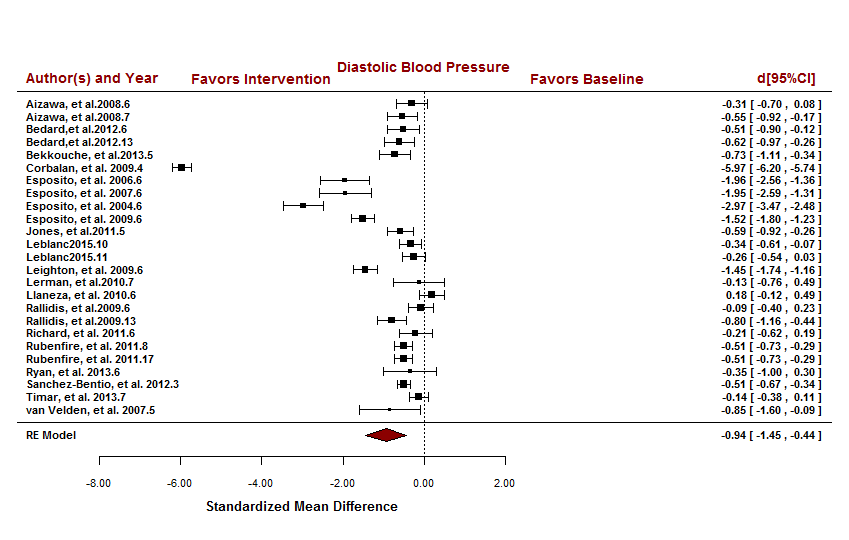


**Figure S6.** Forest Plot for Diastolic Blood Pressure. Note: Squares represent point estimates for each individual study; extended line shows 95% confidence intervals (CIs); dotted line represents the null value of zero; diamond represents the weighted mean effect size for the outcome.


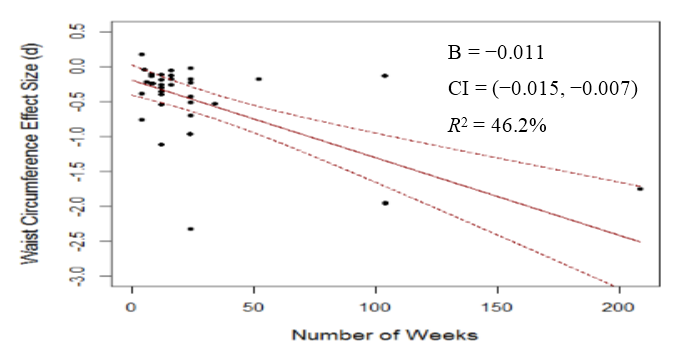


**Figure S7.** Meta-regression Plot for Waist Circumference. Note: Number of weeks of the intervention is represented on the *x*-axis; Outcome of interest is represented on the *y*-axis; B is the unstandardized beta and represents the amount of change in the outcome per week of the intervention; *R*^2^ indicates the percentage of variability accounted for by length.


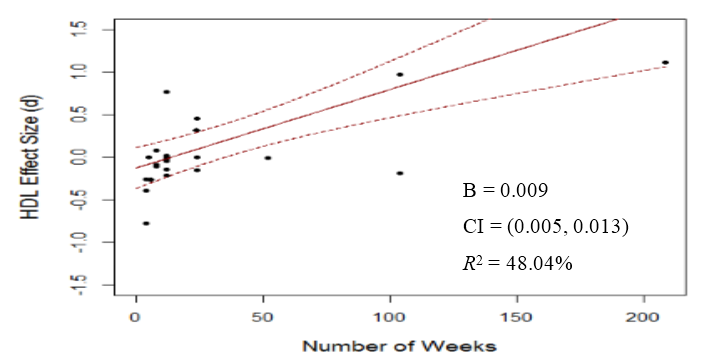


**Figure S8.** Meta-Regression Plot for HDL. Note: Number of weeks of the intervention is represented on the *x*-axis; Outcome of interest is represented on the *y*-axis; B is the unstandardized beta and represents the amount of change in the outcome per week of the intervention; *R*^2^ indicates the percentage of variability accounted for by length.


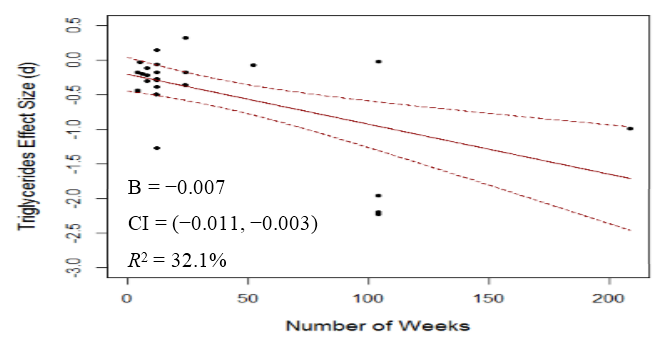


**Figure S9.** Meta-Regression Plot for Triglycerides. Note: Number of weeks of the intervention is represented on the *x*-axis; Outcome of interest is represented on the *y*-axis; B is the unstandardized beta and represents the amount of change in the outcome per week of the intervention; *R*^2^ indicates the percentage of variability accounted for by length.


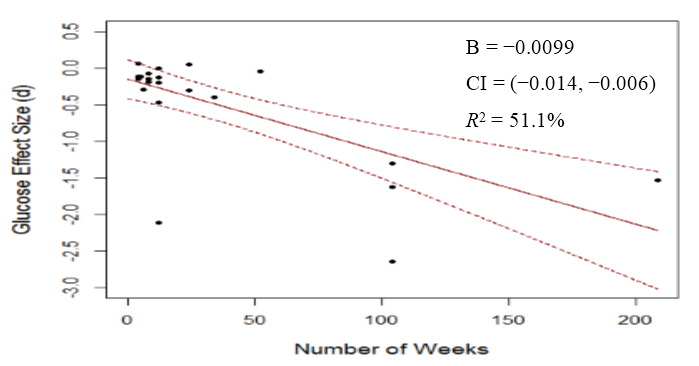


**Figure S10.** Meta-Regression Plot for Fasting Blood Glucose. Note: Number of weeks of the intervention is represented on the *x*-axis; Outcome of interest is represented on the *y*-axis; B is the unstandardized beta and represents the amount of change in the outcome per week of the intervention; *R*^2^ indicates the percentage of variability accounted for by length.


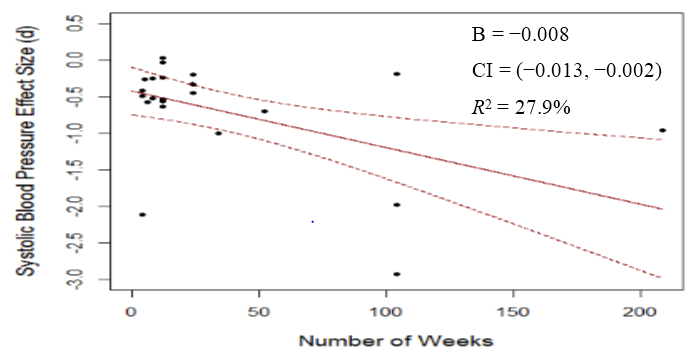


**Figure S11.** Meta-Regression Plot for Systolic Blood Pressure. Note: Number of weeks of the intervention is represented on the *x*-axis; Outcome of interest is represented on the *y*-axis; B is the unstandardized beta and represents the amount of change in the outcome per week of the intervention; *R*^2^ indicates the percentage of variability accounted for by length.

**Figure S12**. Risk of Bias.
